# Effects of moso bamboo encroachment into native, broad-leaved forests on soil carbon and nitrogen pools

**DOI:** 10.1038/srep31480

**Published:** 2016-08-16

**Authors:** Shangbin Bai, Richard T. Conant, Guomo Zhou, Yixiang Wang, Nan Wang, Yanhua Li, Kaiqiang Zhang

**Affiliations:** 1 Jiyang College, Zhejiang A & F University, Zhuji, Zhejiang 311800, PR China; 2 Department of Ecosystem Science and Sustainability, Colorado State University, Fort Collins, CO 80523, USA; 3 Zhejiang Provincial Key Laboratory of Carbon Cycling in Forest Ecosystems and Carbon Sequestration, Zhejiang A & F University, Linan, Zhejiang 311300, PR China; 4 College of Life Sciences, Hainan Normal University, Haikou, Hainan 571158, PR China

## Abstract

Across southern China, Moso bamboo has been encroaching on most neighboring secondary broad-leaved forests and/or coniferous plantations, leading to the land cover changes that alter abiotic and biotic conditions. Little is known about how this conversion alters soil carbon (C) and nitrogen (N). We selected three sites, each with three plots arrayed along the bamboo encroachment pathway: moso bamboo forest (BF); transition zone, mixed forest plots (MF); and broad-leaved forest (BLF), and examined how bamboo encroachment affects soil organic C (SOC), soil total N, microbial biomass C (MBC), microbial biomass N (MBN), water-soluble organic C (WSOC), and water-soluble organic N (WSON) in three forests. Over nine years, moso bamboo encroachment leads to a decrease in SOC and total soil N, an increase in MBC and WSOC, and a decrease in MBN and WSON. Changes in soil C and N occurred mainly in the topsoil. We conclude that moso bamboo encroachment on broadleaved forest not only substantially altered soil C and N pools, but also changed the distribution pattern of C and N in the studied forest soils. Continued bamboo encroachment into evergreen broadleaved forests seems likely to lead to net CO_2_ emissions to the atmosphere as ecosystem C stocks decline.

The soil organic carbon (SOC) and nitrogen (N) pools, an important component of terrestrial carbon and nitrogen pools, play a crucial role in global carbon and nitrogen cycles^1^,^2^. Changes in both SOC and soil N pools that are highly dependent on physico-chemical quality and composition of plant litter could be caused by plant encroachment via altering species composition, and consequently C and N inputs and outputs from the soil^3^. As shifts in soil C and N pools might further potentially influence climate change, the information is critically important that the direction and magnitude of soil C and N changes would be caused by plant encroachment. Most previous studies about the impacts of plant encroachment on soil C and N pools focused on the woody plant species^4^,^5^,^6^, and grass species^7^,^8^. These works have shown that these species altered greatly soil C and N pools in the grasslands and savanna ecosystems, and forest or shrublands. Little quantitative information is available on the influence of native species encroachment or overabundance on soil C and N stocks in the local natural ecosystem. Some native bamboo species became overabundant in their natural distribution range because of the reproduction traits, impacting seriously original forest structure and dynamics^9^, potentially affecting ecosystem functioning and processes (such as C and N cycles). These negative effects are often paid less attention while the overabundant species is economically valuable.

Moso bamboo (*Phyllostachys edulis*) is an economically important plant that is often cultivated for its delicious shoots and versatile culms^10^ and as an important biomass resource across southern China^11^. However, moso bamboo has active rhizomatous clonal growth. The below-ground rhizome system grows laterally, allowing rapid, widespread expansion of ramets^12^. Recently, moso bamoo has encroached on most neighboring secondary broad-leaved forests and/or coniferous plantations, especially in nature reserves^13^,^14^,^15^,^16^. Evergreen broadleaved forest ecosystems are important in regional and global biogeochemical cycles^17^. Changes in these ecosystems caused by plant encroachment can significantly alter components of ecosystem C and N cycles, with potential global consequences for soil and atmospheric chemistry.

Encroachment of moso bamboo is not a result of introduced species; rather it is native to these ecosystems, but it has increased in density and abundance due to changes in abiotic and biotic conditions 18,19. Historically, moso bamboo encroachment into native forests was limited due to shoot and culm harvest in forested areas along field peripheries. Recently-enacted policies prohibiting shoot harvest in nature reserves have increased bamboo encroachment to unprecedented rates, allowing canopy closure within a few years^13^ and often forming nearly monospecific stands. Bamoo encroachment could alter forest floor microclimate with respect to light, temperature, and moisture^18^. This process also substantially reduces tree and shrub diversity in the forests^19^, and modifies soil community structure, and increases microbial biomass and taxonomic diversity^20^.

Little is known about how conversion of broadleaved forests to bamboo-dominated forests alters soil properties and ecosystem storage of carbon (C) and nitrogen (N), although such vegetation changes have important implications for local ecosystem dynamics, as well as regional C and N cycles^21^,^15^. The main aim of this study was to determine soil C and N changes in bamboo forest, bamboo encroachment front area, and unencroached broad-leaved forests to improve our understanding of how moso bamboo encroachment into native, broad-leaved forest affects soil carbon and nitrogen pools.

## Materials and Methods

### Site descriptions

The study area is the Tianmu Mountain National Nature Reserve (119°23′47″-119°28′27″ E,30°18′30″-30°24′55″ N) situated within the northwestern region of Zhejiang Province, in southeastern China. Established in 1956 with the felling ban approval of the Central Government, Tianmu Mountain was promoted as a national reserve in 1986 and included by UNESCO in the International Man and Biosphere Reserve Network in 1996. Tianmu Mountain National Nature Reserve lies in the northern limit of mid-subtropical zone covering a total area of 4,284 ha. The climate there is damp monsoon climate with an annual rainfall of 1390–1870 mm and an annual temperature of 8.8–14.8 °C. It is warmer in winter and cooler in summer than that of other areas in this location. The reserve is one of the sites with the richest subtropical higher plant species in China. There are 2,160 species of higher plants, 1,781 species of seed plants, 151 species of ferns, 222 species of mosses and 69 species of liverworts. Among them, more than 37 species are named after Tianmu Mountain and 1,200 species of medicinal plants. This area boasts a plethora of different kinds of vegetation, such as evergreen and deciduous broadleaved forest, bamboo forest, coniferous forests, marshes and aquatic vegetation. The soils were classified as Ultisols (according to USDA Classification System).

Tianmu Mountain is a famous religious mountain with a long history of Confucianism, Taoism and Buddhism. It has been forbidden to cut any trees and disturb by human activities since established as a nature reserve in 1956. However, the moso bamboo planted originally below the mountain expanded naturally and has expanded from 55.1 ha in 1956 to 105.4 ha in 2014.

### Experimental design

**Figure 1:**
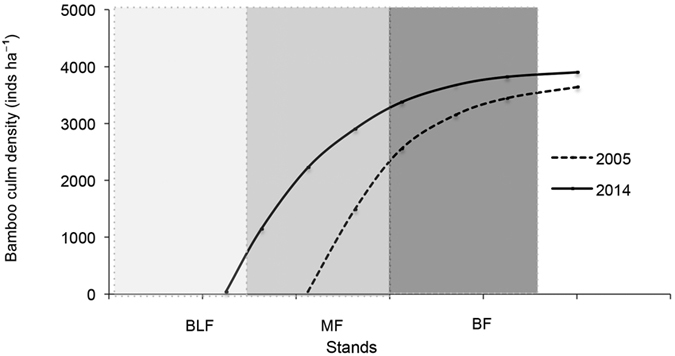
Bamboo culm density in 2005 and 2014 across three contiguous forest zones arrayed along the bamboo encroachment pathway from right to left. The encroachment front moved forward about 9.4 m over 9 years in transition zone (MF). Each point refers to moso bamboo density for corresponding location in each forest zone. The line connecting points together is a smoothing spline. Five replicated samples were collected near the center of each forest plot. BLF: native broad-leaved forest; MF: mixed bamboo and broad-leaved forest; BF: bamboo forest.

In order to quantify the effects of moso bamboo encroachment on soil C and N pools, we employed a complementary chronosequence /transition front approach, i.e., we selected three sites, each with three plots arrayed along the bamboo encroachment pathway: pure moso bamboo forest (BF); transition zone plots that were unencroached plots in 2005, that had been almost fully encroached by 2014 (MF; see Fig. 1); and healthy intact native broad-leaved forest (BLF). The leading edge of the transition zone extends farther into the broad-leaved forest each year, with a well-defined line of moso bamboo. The encroachment front moved forward about 9.4 m over 9 years in transition zone. We monitored the consequence of bamboo encroachment by comparison of soil C and N between BF and BLF, at the same time, in order to further confirm the encroachment consequence and compensate the weakness of the chronosequence method, we compared soil C and N in transition zones (MF) between 2005 and 2014. In addition, we compared soil C and N between 2005 and 2014 in BF and BLF respectively to eliminate mainly the impact of other factors such as climate factors. The BF is unique in having no overstory species besides bamboo. The MF is mainly dominated by the encroaching moso bamboo and *Cyclobalanopsis glauca, Cyclobalanopsis gracilis, Castanopsis sclerophylla, Litsea coreana* Var. *Sinensis*. The BLF is the climax community in this region and is mainly dominated by *Cyclobalanopsis glauca, Cyclobalanopsis gracilis, Castanopsis sclerophylla, Litsea coreana* Var. *Sinensis*. Aspect, slope, elevation, and parent material were consistent within each of the three sites.

Three replicate permanent plots (10 m × 20 m) were established in BF, MF and BLF forest types at each study site in 2005. Five soil cores were collected near the center of each plot using a soil core (diameter 2.5 cm) to 0–60 cm depth and divided into three layers: 0–20, 20–40, and 40–60 cm in each sample site (see Fig. 1). Soil cores were combined in the field, resulting in one composite sample for each plot and a total of nine replicates – one for each of the three plots from all three sites. Samples were weighed to calculate bulk density, and a subsample was dried to constant weight to determine water content. Soil pH was determined with electrometric method in the KCL solution using a soil: liquid suspension ratio of 1:5. The measurements were done in both 2005 and 2014. Table 1 showed soil properties in the plots across three vegetation types in 2005 and 2014.

### Measurements

Soil total organic C (SOC) was measured using the volumetric oxidation method with K_2_Cr_2_O_7_ and titration with ammonium ferrous sulfate^22^ and total nitrogen was measured using the Kjeldahl method^23^. Soil microbial biomass C and N were determined at the beginning of the fast growing season (April 2005 and 2014) using a chloroform fumigation-extraction technique^24^. Microbial biomass C was determined by difference in the amount of C extracted from fumigated and non-fumigated samples, measured with an automated TOC-Vcph Analyzer (Shimadzu, Japan). Microbial biomass N was determined by difference in the amount extractable N from fumigated and non-fumigated samples.

Water-soluble organic C (WSOC) concentration was determined using the methods described by Jiang *et al*.^25^. Briefly, soil WSOC was extracted from 10 g moist soil with addition of 20 mL distilled water. The mixture was shaken for 0.5 h with 250 rpm, and centrifuged for 10 min at 15,000 rpm. The supernatant liquid was filtered through 0.45 μm filterable membrane (Millipore Corp., USA). The C concentration in WSOC was determined using an automated TOC-Vcph Analyzer (Shimadzu, Japan).

Water-soluble organic N (WSON) was extracted from 20 g moist soil with addition of 40 mL distilled water. The mixture was shaken for 0.5 h with 250 rpm, and centrifuged for 10 min at 8,000 rpm. The supernatant liquid was filtered through 0.45 μm filterable membrane (Millipore Corp., USA). Then divided two subsamples. The one subsample was used to determine the total N concentration in the water-soluble organic N by an automated TOC-Vcph Analyzer (Shimadzu, Japan). The other one subsample was used to determine inorganic nitrogen (NH_4_^+^-N and NO_3_^−^-N) concentrations colorimetrically. Water-soluble organic N was determined the amount WSN less extractable inorganic N. The soil C and N pool sizes were calculated respectively by multiplying soil C and N concentrations by the soil bulk density and then multiplying the soil depth.

### Statistical analyses

One-way analysis of variance (ANOVA) (α = 0.05) and least significant difference (LSD) were used to test the differences among forest types for each layer (0–20, 20–40, and 40–60 cm) as well as the 0–60 cm separately, and to test the differences among layers (0–20, 20–40, and 40–60 cm) in the same forest type at each sampling date. Repeated measures analysis of variance (RM ANOVA) was applied to test the differences of measured variables between 2005 and 2014 for each layer as well as the 0–60 cm separately in the same plots with α = 0.05. All statistical analyses were conducted by using SPSS13.0 (Chicago USA).

## Results

### Soil organic carbon and nitrogen changes following moso bamboo encroachment

**Figure 2:**
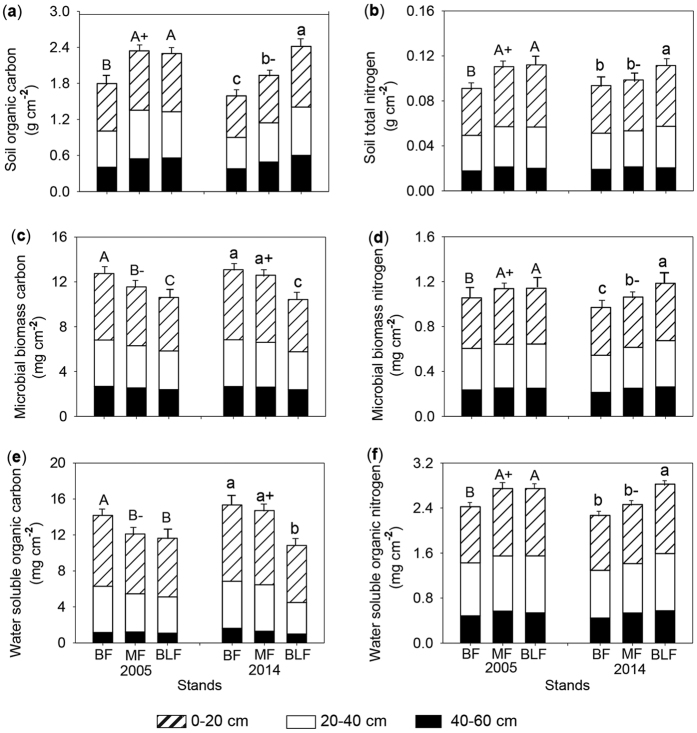
Soil organic C (**a**), Soil total N (**b**), Soil microbial biomass C (**c**), Soil microbial biomass N (**d**), Soil water soluble-organic C (**e**), and Soil water-soluble organic N (**f**) at BF, MF, and BLF in the April of 2005 and 2014. Different uppercase letters indicate significant differences to the total pool size among forests in 2005 (*p* < 0.05); different lowercase letters indicate significant differences to the total pool size among forests in 2014; a plus and a minus sign indicate significant differences to the total pool size between 2005 and 2014 at the same forest.

Encroachment of moso bamboo led to a large reduction of the total amount of soil organic C and N in newly established MF in 2014 relative to the MF that bamboo began encroaching in 2005 (Fig. 2a,b). There was a significant difference between BF and MF, BF and BLF respectively (*P* < 0.05), but no significant difference between MF and BLF in 2005 (*P* > 0.05). Soil organic C was about 30% greater in MF and BLF plots than in BF. Nine years later, SOC decreased significantly in MF (~17%) and did not change significantly for BF and BLF plots. Trends in changes in soil total N was similar to that of SOC, and was about 21% greater in MF and BLF than BF in 2005, but there were no significant differences in 2014 between in MF and BF (*P* > 0.05).

Encroachment of moso bamboo also affected the distribution of soil organic carbon and soil total nitrogen through the soil profiles (Fig. 2a,b). Both SOC and soil total nitrogen decreased with depth in each forest type. There were significant difference between 0–20 cm and 20–40 cm among different forests (*P* < 0.05). SOC at 0–20 cm depth accounted for 44.0%, 42.2%, and 42.2% of the total amount of soil organic C in 2005 in BF, MF, and BLF respectively, and accounted for 43.5%, 41.0%, and 41.8% in 2014. Total soil nitrogen at 0–20 cm depth accounted for 45.7%, 48.4%, and 49.3% of the total amount of total soil N in BF, MF, and BLF respectively, and accounted for 45.3%, 45.8%, and 48.5% in 2014. There was no significant difference in SOC and total soil N at the 40–60 cm depth among different forests (*P* > 0.05).

### Soil microbial biomass C and N changes following moso bamboo encroachment

Details of the soil microbial biomass C concentrations in each forest are listed in Table 2. Changes in soil microbial biomass C and N occurred with conversion of broadleaved forests to moso bamboo forests (Fig. 2c,d), although they comprised a small portion of the total soil organic pools. Soil microbial biomass C increased 9% in MF over time. But it did not differ between in 2005 and 2014 in BF and BLF soils respectively (Fig. 2c,d). There was a significant difference in MBC among different forests (*P* < 0.05). MBC was highest in BF, and lowest in BLF, and about 19% greater in BF than BLF. However compared to 2005, MBN decreased significantly in MF (8%) and BF (7%) plots, and had almost no changes in BLF plot in 2014.

Soil microbial biomass C and N also decreased with increasing soil depth for all three forests. Both were concentrated in the topsoil (0–20 cm) where MBC and MBN made up over 40% of the total amount of MBC and MBN in 0–60 cm. There were significant differences at 0–20 cm depth among different forests (*P* < 0.05). MBC at 0–20 cm depth contributed 46.6%, 45.5%, and 45.0% to the total MBC at 0–60 cm depth in BF, MF, and BLF respectively in 2005 and 47.7%, 47.5% and 44.7% in 2014. However, MBN at 0–40 cm contributed 77.7%, 77.7% and 78.0% to the total MBN at 0–60 cm in BF, MF, and BLF respectively in 2005 and 78.0%, 76.3% and 77.9% in 2014. There were no significant differences in MBC and MBN at the 40–60 cm depth among different forests between 2005 and 2014 (*P* > 0.05). Moso bamboo encroachment altered MBC at 0–20 depth, and altered MBN at 0–40 cm depth.

### Soil water soluble organic C and N changes following moso bamboo encroachment

The total amount of WSOC increased significantly in MF over time, and was 23% greater in 2014 than 2005, but there was no significant difference between 2005 and 2014 in BF and BLF respectively (Fig. 2e). WSOC was greatest in BF, and lowest in BLF in both 2005 and 2014. In contrast to WSOC, soil WSON decreased significantly in MF over time, and was 10% lower in 2014 than 2005, but did not change in BF and BLF. In 2005 there was no difference between MF and BLF, but about 13% higher than in BF. In 2014 there was no difference between MF and BF, but lower than in BLF. Moso bamboo encroachment decreased WSON, but enhanced WSOC.

Although soil WSOC and WSON also showed a decreasing trend with soil depth, WSOC was greater at 0–20 cm depth than at 20–40 cm, but there was no significant difference between 0–20 cm and 20–40 cm for WSON (Fig. 2f). There were significant differences at 0–20 cm for WSOC in MF between 2005 and 2014, but there were no significant differences in BLF and BF between 2005 and 2014. Compared with 2005, WSON at 0–20 cm depth decreased quickly in MF in 2014, but did not alter in BF and BLF in 2014.

## Discussion

Encroachment of bamboo on native evergreen broadleaved forests decreased soil pools of both C and N. This finding is consistent with results from other nature reserves, where sites encroached by moso bamboo have been compared with nonencroached sites^15^. The difference in soil C and N pools between sites with and without bamboo increased over time, indicating that the net C and N reduction were induced by the encroaching bamboo. This decrease in soil C stocks was coupled with increasing soil respiration, which is 25% greater in bamboo forest than broadleaved forest in Jinyun^26^, and 98% greater than evergreen broadleaved forest in Tianmu mountain nature reserve^27^. These observations suggest that decomposition rates increased with bamboo encroachment. Increased decomposition rates might be caused by changes in soil microbial composition^20^, and the increased significantly microbial biomass with bamboo encroachment. The soil C and N stocks in native broadleaved forests built up over long period of time before bamboo encroachment began^28^,^29^. The decrease in native broadleaved forest soil suggests that bamboo encroachment can rapidly alter soil C and N dynamics.

Soil organic C and total N decreases after 9-years of bamboo encroachment are consistent with other examples of grass and woody encroachment on native ecosystems^6^,^30^. But our results are not consistent with result from some ecosystems that were encroached by different species, such as N-fixing species^4^. While N-fixing species usually increase N into ecosystems, higher nitrogen content in moso bamboo^31^,^32^ suggests that moso bamboo consumes a lot of nitrogen due to its fast growth rate, leading to soil N depletion. The decline of SOC may be related to the increase in pH (Table 1), which is consistent with the results of other studies^33^, suggesting that soil pH affected the regulation of the decomposition of fresh organic matter considerably along with SOM decomposition by influencing microbial activity^34^. Furthermore, the increasing bulk density with bamboo encroachment (Table 1) might be attributed to the decreased SOC, the weakened aggregation and consequent decrease in the volume of micropores.

Microbial biomass C and N are active pools of organic matter in soils. The increased MBC with moso bamboo encroachment (Fig. 2c) might be attributed to the soil microbial communities and diversity modified by bamboo invasion^20^. The increased amount and activity of microbial communities further promote organic matter decomposition, accelerating soil respiration rates, and subsequently SOC reduction. Wardle^35^ confirmed that lower pH could accelerate microbial biomass turnover and higher pH reduced microbial biomass turnover rate, extending turnover time. In this study, the pH increased with bamboo encroachment, suggesting that more C fixed into the microbial body temporarily through assimilation to make MBC increase. Additionally, the increase of fungi root colonization caused by bamboo encroachment^36^,^37^ may also be a reason for the increase in microbial biomass carbon.

Soil WSOC has been found to be mainly released from litter decomposition, root exudation, and mineralization of soil organic matter through microbial activities^38^. Moso bamboo altered the rate and type of litter input because of changes in species composition^19^, and subsequently WSOC concentrations. In this study, the increased WSOC with bamboo encroachment indicated organic matter decomposition accelerated and soil respiration rates enhanced, reducing the SOC and TN. In compare to WSOC, soil WSON decreased with bamboo encroachment (Fig. 2f). Although WSON is a small portion of soil organic N, the importance of WSON in meeting plant N requirement has recently been emphasized^39^,^40^ and can be an important N source for some plants^39^. Fast-growing bamboo uptake of small molecule WSON might be part of the reason for the decrease of WSON in BF and MF (Fig. 2f). Lower soil pH could lead to increased WSON concentrations^41^, while increased pH in BF and MF might contribute to WSON reduction.

Encroachment of moso bamboo on broadleaved forest not only substantially decreased soil organic carbon and soil nitrogen, but also affected depth profiles. There were significant topsoil differences in different forests, suggesting the effects of moso bamboo encroachment on soil carbon and soil nitrogen occurred mainly in the topsoil (0–20 cm, and 20–40 cm). Moso bamboo encroachment altered plant diversity^19^, especially tree and shrub diversity. Loss of plant diversity could drive a decrease in C inputs into the topsoil and C accumulation in soil^42^,^43^. Moreover, due to lower C and higher N contents in litter of *P. pubescens* than those of broadleaved trees^31^,^32^, the decomposition of litter in the MF and BF occurs more quickly in the topsoil, causing soil carbon and nitrogen reduction in the topsoil. Moso bamboo roots are mainly distributed in the surface 40 cm^44^, and root growth and exudation could affect microorganism growth and production, altering soil C and N at 0–40 cm depth. Moso bamboo propagates clonally by its rhizomes. Mature bamboo plants close to the forest boundary produce new rhizomes typically at a depth of 15–40 cm in the soil. Only some of them spread into neighboring forest, so bamboo encroachment is spatially patchy rather than uniform across the landscape. Thus the encroached evergreen broadleaved forests will be gradually dominated by bamboo with mixtures of different ages and canopy structures. As bamboo density increases, the effect of encroachment on soil C and N would be likely greater in high-density areas.

Decreased soil C and N pools in response to bamboo encroachment are another example of plant invasion having a detrimental effect on ecosystems^29^,^45^. Moso bamboo has been expanding every year, leading to decreases in broadleaved evergreen forest area. If encroachment is not contained, the protected forests adjacent to moso bamboo in Tianmushan Nature Reserve and other nature reserves may be dominated by moso bamboo forest and lose much of their conservation value. At the same time, moso bamboo forests cover about 3.37 million ha, about 2% of the total Chinese forest area and about 70% of the total Chinese bamboo area^46^. It is mainly distributed in southern China, including 12 provinces such as Fujian, Jiangxi, Zhejiang, etc. If the rapid expansion of moso bamboo forest into adjacent broadleaved forests continues in these subtropical areas, regional decrease of broadleaved forest C will continue to contribute to increasing atmospheric CO_2_ concentration.

## Conclusion

Both C and N pools in the soil decreased along gradients of bamboo cover, suggesting that continued growth and C storage in this broadleaved forest ecosystems are likely to be constrained by moso bamboo encroachment. Despite the fact that bamboo is a fast-growing species that fixes C at a high rate, bamboo encroachment leads to a significant decline in soil C over time. If our findings also occur in other bamboo encroachment areas, widespread increases in the cover of bamboo in areas formerly dominated by evergreen broadleaved tree species will lead to a decline in regional C stocks in the southern China. Measurements taken to prevent bamboo from encroaching into evergreen broadleaved forests, could offer C retention and thus climate mitigation benefits.

## Additional Information

**How to cite this article**: Bai, S. *et al*. Effects of moso bamboo encroachment into native, broad-leaved forests on soil carbon and nitrogen pools. *Sci. Rep.* **6**, 31480; doi: 10.1038/srep31480 (2016).

## Figures and Tables

**Table 1:** Soil properties of the 0–60 cm depth in the plots across three forest types in 2005 and 2014.

Time	Stands	Soil layer(cm)	Bulk density(g cm^−3^)	Water content (%)	pH value
		0–20	1.01 a B	31.3A−	5.19 a
	BF	20–40	1.30 a A	30.1 b A−	5.15 a
		40–60	1.35A	27.2 b B	5.16 a
		0–20	0.95 b B	32.2 B−	5.02 b A
2005	MF	20–40	1.22 b A	33.4 a A	4.71 b B−
		40–60	1.32A	31.6 a B+	4.67 b B−
		0–20	0.93 b B	30.8A−	4.65 c
	BLF	20–40	1.20 b A	29.1 c B−	4.58 b
		40–60	1.31A	28.4 b B	4.61 b
		0–20	1.01 a B	32.9 b A+	5.21 a
	BF	20–40	1.29A	32.0 b A+	5.17 a
		40–60	1.34A	28.3 B	5.14 a
		0–20	0.98 a B	34.3 a A+	4.91 b
2014	MF	20–40	1.27A	33.1 a A	5.03 b +
		40–60	1.34A	28.8 B−	5.00 b +
		0–20	0.93 b B	33.4 abA+	4.72 c
	BLF	20–40	1.21A	31.6 b B+	4.67 c
		40–60	1.31A	29.0C	4.65 c

BF: bamboo forest; MF: mixed bamboo and broad-leaved forest; BLF: native broad-leaved forest.

Figures followed by different lowercase letters in the same column indicate significant differences among forests within each soil layer in the same year at 0.05 level; different uppercase letters in the same column indicate significant differences among different soil layers within each forest in the same year at 0.05 level; a plus and a minus sign in the same column indicate significant differences between 2005 and 2014 within each soil layer at the same forest at 0.05 level.

**Table 2:** Soil carbon and nitrogen contents in the topsoil of different stands in 2005 and 2014.

	2005 BF	2005 MF	2005 BLF	2014 BF	2014 MF	2014 BLF
TOC (g kg^−1^)	39.15a	50.98bA	52.31b	37.23a	40.37aB	54.18b
MBC (mg kg^−1^)	293.71a	266.40bA	256.46c	299.19a	280.00aB	250.46c
WSOC (mg kg^−1^)	410.32a	370.78bA	350.12c	420.00a	421.10aB	340.86b
TN (g kg^−1^)	2.06a	2.81bA	2.97b	2.10a	2.31aB	2.90b

Note: figures followed by different lowercase letters in the same row indicate significant differences among forests in the same year at 0.05 level; different uppercase letters in the same row indicate significant differences between in 2005 and 2014 in the same forests at 0.05 level.
